# Mental disorders pattern in staff of a military unit in Iran: the role of metabolic syndrome on latent class membership

**DOI:** 10.1186/s12888-021-03537-z

**Published:** 2021-10-19

**Authors:** Abbas Abbasi-Ghahramanloo, Mohammadkarim Bahadori, Esfandiar Azad, Nooredin Dopeykar, Parisa Mahdizadeh, Amir Vahedian Azimi, Hossein Amini

**Affiliations:** 1 Health Management Research Center, Baqiyatallah University of Medical Sciences, Tehran, Iran; 2 Department of Public Health, School of Health, Ardabil University of Medical Sciences, Ardabil, Iran; 3 Behavioral sciences research center, lifestyle institute, Baqiyatallah University of Medical Sciences, Tehran, Iran; 4 Trauma research center, Nursing Faculty, Baqiyatallah University of Medical Sciences, Tehran, Iran

**Keywords:** Mental disorders, Comorbidity, Latent class analysis, Staff, Military unit

## Abstract

**Introduction:**

Mental disorders are among the most prevalent health problems of the adult population in the world. This study aimed to identify the subgroups of staff based on mental disorders and assess the independent role of metabolic syndrome (MetS) on the membership of participants in each latent class.

**Methods:**

This cross-sectional study was conducted among 694 staff of a military unit in Tehran in 2017. All staff of this military unit was invited to participate in this study. The collected data included demographic characteristics, anthropometric measures, blood pressure, biochemical parameters, and mental disorders. We performed latent class analysis using a procedure for latent class analysis (PROC LCA) in SAS to identify class membership of mental disorders using Symptom Checklist-90.

**Results:**

Three latent classes were identified as healthy (92.7%), mild (4.9%), and severe (2.4%) mental disorders. Having higher age significantly decreased the odds of belonging to the mild class (adjusted OR (aOR = 0.21; 95% confidence interval (CI): 0.05–0.83) compared to the healthy class. Also, obesity decreased the odds of membership in mild class (aOR = 0.10, 95% CI: 0.01–0.92) compared to healthy class. On the other hand, being female increased the odds of being in severe class (aOR = 9.76; 95% CI: 1.35–70.65) class in comparison to healthy class.

**Conclusion:**

This study revealed that 7.3% of staff fell under mild and severe classes. Considering educational workshops in the workplace about mental disorders could be effective in enhancing staff’s knowledge of these disorders. Also, treatment of comorbid mental disorders may help reduce their prevalence and comorbidity.

## Introduction

Mental disorders are among the most prevalent health problems of the adult population in the world [1]. According to estimations of Burden of Disease Study 2015 (GBD 2015), seven of the top 25 causes of years lived with disability (YLD) globally were mental disorders, with major depressive disorder rated as 2dn and anxiety disorders rated as 9th grade [2]. Also, mental disorders were the second leading cause of disease burden in terms of YLD and the sixth leading cause of disability-adjusted life years (DALYs) in the world in 2017 [3].

Estimates of last year’s prevalence of mental health disorders vary between 9.6 and 27.8% in the general adult population [4–9]. Studies from Western countries estimated that at least one-fourth of the adult population in these countries meet the criteria of at least one mental disorder during 12 months [4, 10–12]. Various national studies have already been conducted on the prevalence of mental health disorders in Iran [13–16]. The prevalence of these disorders was reported from 17.1 to 23.6% in large national studies in Iran. A recent meta-analysis indicated that the prevalence of mental disorders in studies that used screening tools was 31.03% and in studies that used clinical interviews was 25.42% among Iranian people [17].

Comorbidity between different mental disorders is extensive with up to 50% of those who have one mental disorder also having at least one additional comorbid mental disorder [5, 8, 18]. Mental disorder’s comorbidity seems to a large degree to be due to common liability factors for different disorders [19, 20]. Also, comorbidity is related to severity and chronicity [21–23]. However, most studies have been focused on the prevalence estimation of mental disorders in different countries. Estimates of comorbidity may increase our understanding of the potential effects on common mental disorders [24].

It is documented that patients with mental disorders such as schizophrenia and bipolar disorder have an increased prevalence of MetS and its components [25]. A recent meta-analysis indicated that the prevalence of MetS is 58% higher in psychiatric patients than in the general population [26]. Consequently, it is suggested that MetS is general comorbidity seen in the different psychiatric patient groups [27]. However, there is limited information about its effect on different subtypes of mental disorders.

Based on the above-mentioned background, this study aimed to identify the subgroups of staff based on mental disorders and assess the independent role of MetS on the membership of participants in each latent class.

## Methods

This cross-sectional study was conducted in the framework of health monitoring of a military unit. This study was performed on 694 staff in Tehran in 2017. All staff of this military unit was invited to participate in this study. All staff with military affiliation were eligible to participate in this study and staff without any military affiliation were excluded from this study. All experimental protocols were approved by Ethics Committee of Baqiyatallah University of Medical Sciences and all methods were carried out in accordance with Declaration of Helsinki. Permission to conduct the study was obtained from this Committee of and all staff had signed an informed consent form.

The collected data included demographic, anthropometric, blood pressure measurement, biochemical parameters, and mental disorders. Questions on demographic characteristics included age, sex, marital status, history of working, and so on. Anthropometric measures were weight (kg), height, and WC (cm). For body mass index participants were divided into the following groups: low weight (BMI < 18.5 kg/m2), normal weight (BMI = 18.5–24.9 kg/m2), overweight (BMI =25–29.9 kg/m2), and obesity (BMI ≥ kg/m2). Also, the following blood biochemical parameters were used as components of MetS: fasting plasma glucose (FPG), triglycerides (TG), and high-density lipoprotein cholesterol (HDL-C) [28, 29].

In this study, the International Diabetes Federation (IDF) definition was used to determine the MetS status for participants. According to this definition, abdominal obesity is shown as WC ≥ 94 cm in males and ≥ 80 in females. Elevated triglycerides is another component of MetS (≥ 150 mg/dl). In addition reduced HDL-C is another frequent component of MetS (< 40 mg/dl in males vs. < 50 mg/dl in females). Elevated blood pressure (systolic BP ≥ 130 mmHg and diastolic BP ≥ 85 mmHg) or treatment of prior diagnosed elevated blood pressure was considered as other component of MetS. Finally, fasting glucose (≥ 100 mg/dl) or previous detection of type 2 diabetes [29].

Mental disorder status was measured using the Symptom Checklist-90 (SCL-90). The SCL-90 is a multidimensional self-report measure, assessing the severity of current mental disorders. It assesses nine disorder dimensions: hostility, anxiety, OCD, interpersonal sensitivity, somatization, psychoticism, paranoid ideation, depression, and phobic anxiety. These scale items were rated on a 0-to-4 response scale from 0 (not at all) to 4 (extremely) specifying how much each has bothered them during the past 7 days [30, 31]. Derogatis et al. assessed a new self-report symptom inventory for the first time. This inventory is named the SCL-90. In this study, a sample of 90 symptomatic volunteers served as subjects and was administrated both the SCL-90 and the MMPI. Comparisons of the nine primary symptoms dimensions of the SCL-90 with the set of MMPI scales reflected very high convergent validity for the SCL-90 in this study [30]. Also, in Iran, Anisi et al. assessed the validity and reliability of SCL-90 among the staff of a military unit. They found that the range of internal consistency coefficient (Cronbach’s alpha) of SCL-90 subscales was from 0.75 to 0.90. Also, the test-retest consistency coefficient of subscales ranged from 0.57 to 0.90 [31].

Standard T scores were used to categorize individuals in each dimension. In each dimension scores between 40 and 60 were considered normal. Also, scores between 61 and 70 were considered as mild, scores of 71–80 were considered as moderate, and scores higher than 80 were considered as severe [31]. In our analysis, for all dimensions, scores higher than 60 were considered as having the disorders.

We used simple descriptive statistics to investigate the characteristics of staff and the level of engagement in each psychological disorder. Then, we performed the Latent class analysis (LCA) six times, using one to seven classes to identify the best model that can fit the data [32]. LCA is a person-centered approach that uses categorical and cross-sectional observed variables to find subtypes of related cases and yield categorical latent classes of participants [33]. Understanding of comorbidity of mental disorders is possible with subgrouping of subjects based on these disorders.

LCA is a cross-sectional latent variable mixture modelling approach. Like all latent variable mixture modelling approaches, LCA aims to find heterogeneity within the study population. This is done by analysing an individual’s patterns of behaviours, such as mental health indicators, and finding common types, called classes. Each individual is probabilistically assigned to a class. That results in subgroups of individuals, who are most similar to each other and most distinct from those in other classes [34, 35].

For model identification, we fitted each model 20 times using different starting values. To select the best model, we calculated and compared the likelihood-ratio statistic G2, the Akaike information criterion (AIC), the Bayesian information criterion (BIC), Entropy, and the log-likelihood values across six models. Among these indices, lower values of G2, AIC, BIC, and the log-likelihood and higher value of Entropy showed a more optimal model fit. In addition to these indices, the interpretability and parsimony of a model could help in the selection of the final model. Classes identified in the model should be meaningful, and a simpler model is preferable [36].

Nine dichotomous observable variables were used for subgrouping of the staff based on psychological disorders. These variables were hostility, anxiety, OCD, interpersonal sensitivity, somatization, psychoticism, paranoid ideation, depression, and phobic anxiety. After identifying the optimal model (three-class model in this study), we conducted an LCA with covariates to detect the effect of predictors of latent class membership. To this end, we included five variables in the analysis that all of which were dummy coded variables. These variables were age, sex, marital status, BMI, and MetS status. The “healthy” class was considered as the reference class when investigating predictors of class membership.

Simple statistical analysis, chi-square tests were used with SPSS 16. LCA was performed by using PROC LCA in SAS 9.2 software. In all analyses, *P*-value < 0.05 was considered statistically significant.

## Results

Out of 720 staff, a total of 694 were participated in the study and returned the questionnaires to the researchers (response rate: 93.39%). This study indicated that the mean age of the participants was 45.43 ± 7.96 (range: 27–69) years. Table 1 indicates that among all participants, 539(77.7%) of them were male and only 38(5.5%) were single. Also, 125(18.0%) of them were obese and 247(35.6%) had MetS.

**Table 1:** Demographic characteristics of study population

Items	N	%
**Age**		
< 50	273	67.9
≥ 50	129	18.6
**Sex**		
Male	539	77.7
Female	144	20.7
**Marital status**		
Single	38	5.5
Married	584	84.1
**BMI**		
< 30	490	79.7
≥ 30	125	18.0
**MetS**		
No	407	58.6
Yes	247	35.6

The prevalence of each mental disorder is shown in Table 2. The results suggest that the prevalence of OCD was higher than other disorders. Also, the prevalence of phobic anxiety had the lowest prevalence. Table 2 also presents the conditional distribution of MetS status at each level of the mental disorders. Table 3 shows different measures of model selection for classes one to six. According to model selection criteria and interpretability of the results, the three-class model was chosen for the subgrouping of the staff. It should be noted that the two-class model had the lowest value of BIC and the three-class model had the lowest AIC.

**Table 2:** Mental disorders status by MetS among staff of a military unit

Items	Total (694)	MetS		***P***-value
	N (%)	No N (%)	Yes N (%)	
**Hostility**				
No	633(91.2)	368(97.1)	232(96.7)	0.762
Yes	21(3.0)	11(2.9)	8(3.3)	
**Anxiety**				
No	634(91.4)	364(96.0)	236(98.3)	0.107
Yes	20(2.9)	15(4.0)	4(1.7)	
**OCD**				
No	620(89.3)	359(94.7)	227(94.6)	0.940
Yes	34(4.9)	20(5.3)	13(5.4)	
**Interpersonal sensitivity**				
No	632(91.1)	364(96.0)	234(97.5)	0.329
Yes	22(3.2)	15(4.0)	6(2.5)	
**Somatization**				
No	632(91.1)	366(96.6)	233(97.1)	0.725
Yes	22(3.2)	13(3.4)	7(2.9)	
**Psychoticism**				
No	631(90.9)	362(95.5)	235(97.9)	0.116
Yes	23(3.3)	17(4.5)	5(2.1)	
**Paranoid ideation**				
No	629(90.6)	362(95.5)	232(96.7)	0.478
Yes	25(3.6)	17(4.5)	8(3.3)	
**Depression**				
No	629(90.6)	359(94.7)	235(97.9)	0.049
Yes	25(3.6)	20(5.3)	5(2.1)	
**Phobic anxiety**				
No	636(91.6)	366(96.6)	237(98.8)	0.096
Yes	18(2.6)	13(3.4)	3(1.3)

**Table 3:** Comparison of LCA Models With Different Latent Classes Based on Model Selection Statistics

Number of latent class	Number of parameters estimated	G^2^	df	AIC	BIC	Entropy	Maximum log-likelihood
1	9	820.69	502	838.45	878.80	–	− 902.69
2	19	188.50	492	226.50	311.68	0.99	− 586.72
**3**	**29**	**134.98**	**482**	**192.98**	**323.00**	**0.94**	**− 559.96**
4	39	110.08	472	188.08	362.92	0.92	− 547.51
5	49	93.76	462	191.76	411.43	0.93	−539.34
6	59	78.42	452	196.42	460.92	0.94	− 531.68

*LCA* latent class analysis, *AIC* Akaike information criterion, *BIC* Bayesian information criterion

Table 4 presents the three-class latent class model. Participants of this study were grouped into the “healthy” class (92.7%), “mild” class (4.9%), and “severe” class (2.4%). Specifically, staff in the “healthy” class had near-zero probabilities of having any mental disorders. Participants in the “mild” class had elevated probabilities for some disorders (i.e. OCD, paranoid ideation, and depression). However, there is no above 50% probability in this class. Finally, staff in “severe” class had a high probability of engaging in all mental disorders. The probability of having all disorders was high among the staff of this class.

**Table 4:** The three latent class model of mental disorders patterns among staff of a military unit

	Latent class		
	Healthy	Mild	Severe
**Latent class prevalence**	0.927	0.049	0.024
**Item-response probabilities**			
Hostility	0.014	0.080	**0.616**
Anxiety	0.000	0.153	**0.909**
OCD	0.015	0.346	**0.865**
Interpersonal sensitivity	0.000	0.249	**0.862**
Somatization	0.010	0.184	**0.610**
Psychoticism	0.002	0.187	**0.975**
Paranoid ideation	0.007	0.316	**0.671**
Depression	0.003	0.299	**0.851**
Phobic anxiety	0.004	0.173	**0.626**

The probability of a “No” response can be calculated by subtracting the item-response probabilities shown above from 1

^*^ Item-response probabilities >.5 in bold to facilitate interpretation

We found three significant predictors of latent class membership (Table 5), implying different distribution of latent class membership across these factors. Higher age (50 years old and higher) significantly decreased the odds of being in mild class (OR = 0.21, 95% CI: 0.05–0.83) compared to healthy class. Similarly having a higher BMI (30 and more) significantly decreased the odds of membership in mild class (OR = 0.10, 95% CI: 0.01–0.92) in comparison to the healthy class. On other hand, being female, compared to being male, increased the odds of membership in severe class (OR = 9.76, 95% CI: 1.35–70.65) compared to healthy class. The results of the present study indicated that marital status and having MetS did not show a significant effect on the membership of staff in different classes.

**Table 5:** Predictors of membership in latent classes of mental disorders patterns among staff of a military unit

Predictors	***P-***value	Healthy	Mild	Severe
		OR(95%CI)	OR(95%CI)	OR(95%CI)
Age (50 and higher)	0.0175	**Reference**	**0.21(0.05–0.83)**	**0.47(0.08–2.72)**
Sex (being female)	0.0033	**Reference**	**0.11(0.01–1.20)**	**9.76(1.35–70.65)**
Marital status (being single)	0.1235	**Reference**	**1.04(0.19–5.61)**	**0.10(0.00–15.77)**
BMI (30 and higher)	0.0462	**Reference**	**0.10(0.01–0.92)**	**1.73(0.35–8.43)**
Having MetS	0.1839	**Reference**	**1.46(0.59–4.59)**	**1.03(0.14–7.41)**

## Discussion

The results of this study indicated the prevalence of each mental disorder namely, hostility, anxiety, OCD, interpersonal sensitivity, somatization, psychoticism, paranoid ideation, depression, and Phobic anxiety. OCD was a common disorder with a rate of prevalence of 4.9% among participants of this study. The order of most prevalent mental disorders varies in different countries. For example in Europe [4] any anxiety disorder, anxiety disorders in China [37], and mood disorders in Latvia [38] were the most common disorders. The Iranian Mental Health Survey showed that the most prevalent group of disorders among Iranian adults was the group of anxiety disorders (15.6%). Also, this study found that the most prevalent particular DSM-IV disorder was major depressive disorder (12.7%), followed by generalized anxiety disorder (5.2%), and OCD (5.1%) [15]. Noorbala et al. in a national study revealed that the prevalence of anxiety and somatization symptoms was higher than social dysfunction and depressive symptoms in Iranian adults [16]. Because of using different diagnostic tests and also different cut-offs the results of the present study aren’t comparable with national estimates of mental disorders. However, it should be noted that before employment, all staff of military units, should pass several physical and mental tests. Employment in these units needs to acquire an acceptable score. With considering this important point, the differences in the pattern of mental disorders in the staff of military units with other people are to be expected.

In this study, we identified different patterns of mental disorders with LCA and were able to detect three distinct classes that we named as follows: healthy, mild, and severe. The probability of engaging in each mental disorder is quite low among the staff in latent class 1. In the second class, although there are no above 50% probabilities, however, some disorders have an elevated probability of occurring. Finally, in the third class, the probability of all disorders is quite high among the participants. Understanding patterns of comorbidity within mental disorders is essential to the understanding influence of mental disorders on premature mortality and the contribution of these disorders to the global burden of disease [39, 40]. Comorbidity within mental disorders is pervasive and the risk persists over time [41]. Our findings indicated that among 2.4% of the participants, there is comorbidity among different mental disorders. This result is broadly consistent with those of other comprehensive studies of comorbidity within mental disorders [5, 41–44]. For example, a big study from 27 countries concluded that each prior lifetime mental disorder was associated with an increased risk of subsequent first onset of each other disorder [45]. The presence of different disorders at the same time is extremely common in the realm of psychopathology [5]. Anyway, comorbidity of mental disorders has received considerable attention in the clinical literature, because individuals with comorbid mental disorders have a poorer prognosis, worse treatment outcomes, and higher suicide rates [46, 47]. In addition, mental disorder’s comorbidity is associated with an increased risk of the onset of a wide range of chronic physical conditions [48]. As a result, this condition may have potential impacts on staff (e.g., discrimination), organizations (e.g. loss of productivity), workplace health and compensation authorities (e.g. rising job stress-related claims), and social welfare system (e.g. rising working-age disability pensions for mental disorders) [49].

To the best of our knowledge, this study is the first attempt to use LCA for subgrouping of staff based on mental disorders in Iran. There are some studies from other countries that have employed this approach among specific groups. Although different studies used various indicators for subgrouping of other populations than staff, some of the relatively similar ones will be discussed below:

Villaobos-Gallegos et al. found five separated subgroups of psychiatric symptoms in a sample of patients with co-occurring disorders, which were labeled as follow: mild, mild-moderate, moderate, moderate-severe, and severe [50]. Tsaai et al. were able to identify three latent classes for psychiatric comorbidity among adults with schizophrenia, including sole schizophrenia, comorbid anxiety and depressive disorders with schizophrenia, and comorbid addiction and schizophrenia [51]. In a population-based study, the authors identified four latent classes of depressive symptoms among respondents with anxiety. These classes are named as severe typical, not depressed, moderate typical, and mild typical [52]. The literature review indicates that studies obtained solutions with the quantitative and qualitative difference between classes, suggesting that subgroups are mostly based on combinations of specific disorders and symptoms severity. Similar to our findings and despite methodological and sample differences (i.e. indicators were categorical), mental disorders may be distributed across distinctive levels of severity.

Some studies examined the association between obesity and mental disorders in different age groups. Bruffaerts et al. indicated that obese individuals are more likely to have a mood disorder or more than one mental disorder [53]. Another study showed that compared to normal-weight peers, adolescents with overweight or obesity reported psychological problems and suicidal thoughts more often [54]. Our findings showed that being obese decreases the odds of membership in mild class compared to healthy class. It should be noted that in this study we did not assess the status of taking psychotropic medications or medications that are known to be associated with weight gain. With considering this possible source of bias and because of the borderline *p*-value (i.e. 0.0462) and lack of evidence in the Iranian population, more investigations are needed to assess the association between obesity and mental disorders.

Previous studies attempted to assess the association between MetS and mental disorders [25–27]. Although most of them showed the association between having MetS and high odds of engaging in mental disorders, however in the present study having MetS did not have a significant effect on the membership of participants in latent classes of mental disorders.

This study has some limitations. First, the study was conducted in only one military unit in Iran; therefore, it may not be generalizable to other parts of Iran. Second, data were self-reported about mental disorders and might be subject to recall, response, or other possible biases. Third, due to the cross-sectional design of the study, the causal inference could not be identified based on our findings.

## Conclusion

This study revealed that a large percentage of the staff fell under latent class of healthy. However, some staff belonged to sever class. In the severe class, the probability of engaging in all disorders is quite high in this class. We found that age and BMI were associated with mild class and only sex was associated with the severe class. Our findings highlight a need for targeted intervention and treatment designs in order to reduce mental disorder’s prevalence and comorbidity. Also, some educational workshops in the workplace about mental disorders could be effective in enhancing staff’s knowledge toward these disorders.

## Data Availability

The datasets generated during the current study are not publicly available since they will contain patient data and the informed consent agreement does not include sharing data publicly. However, the data are available from the corresponding author on reasonable request.

## Abbreviations

LCA: latent class analysis
AIC: Akaike Information Criterion
BIC: Bayesian Information Criterion
